# *trans*-4-(Phenoxy­meth­yl)cyclo­hexane­carboxylic acid

**DOI:** 10.1107/S1600536808007381

**Published:** 2008-03-29

**Authors:** Jun Yang, Qing-Rong Qi, Wen-Cai Huang, Hu Zheng

**Affiliations:** a Department of Medicinal Chemistry, West China School of Pharmacy, Sichuan University, Chengdu 610041, People’s Republic of China; b Department of Pharmaceuticals and Bioengineering, School of Chemical Engineering, Sichuan University, Chengdu 610065, People’s Republic of China

## Abstract

The title compound, C_14_H_18_O_3_, is an important model compound in the synthesis of phenolic ethers. The cyclo­hexane ring adopts a chair conformation. In the crystal structure, adjacent mol­ecules are linked by O—H⋯O hydrogen bonds.

## Related literature

For related literature, see: Dunitz & Strickler (1966 ▶); Sekera & Marvel (1933 ▶); Luger *et al.* (1972 ▶).
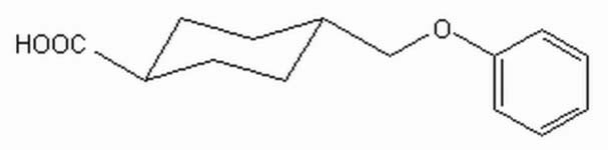


## Experimental

### 

#### Crystal data

C_14_H_18_O_3_
*M* *_r_* = 234.28
Monoclinic, 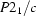

*a* = 6.178 (3) Å
*b* = 35.042 (8) Å
*c* = 6.526 (3) Å
β = 113.93 (4)°
*V* = 1291.4 (9) Å^3^
*Z* = 4
Mo *K*α radiation
μ = 0.08 mm^−1^
*T* = 292 (2) K
0.45 × 0.25 × 0.24 mm

#### Data collection

Enraf–Nonius CAD-4 diffractometer
Absorption correction: none
2657 measured reflections
2330 independent reflections
1301 reflections with *I* > 2σ(*I*)
*R*_int_ = 0.001
3 standard reflections every 250 reflections intensity decay: 1.8%

#### Refinement

*R*[*F* ^2^ > 2σ(*F* ^2^)] = 0.072
*wR*(*F* ^2^) = 0.149
*S* = 0.97
2330 reflections
156 parameters
9 restraints
H-atom parameters constrained
Δρ_max_ = 0.17 e Å^−3^
Δρ_min_ = −0.17 e Å^−3^

### 

Data collection: *DIFRAC* (Gabe *et al.*, 1993 ▶); cell refinement: *DIFRAC*; data reduction: *NRCVAX* (Gabe *et al.*, 1989 ▶); program(s) used to solve structure: *SHELXS97* (Sheldrick, 2008 ▶); program(s) used to refine structure: *SHELXL97* (Sheldrick, 2008 ▶); molecular graphics: *ORTEP-3 for Windows* (Farrugia, 1997 ▶); software used to prepare material for publication: *SHELXL97*.

## Supplementary Material

Crystal structure: contains datablocks global, I. DOI: 10.1107/S1600536808007381/bv2092sup1.cif

Structure factors: contains datablocks I. DOI: 10.1107/S1600536808007381/bv2092Isup2.hkl

Additional supplementary materials:  crystallographic information; 3D view; checkCIF report

## Figures and Tables

**Table 1:** Hydrogen-bond geometry (Å, °)

*D*—H⋯*A*	*D*—H	H⋯*A*	*D*⋯*A*	*D*—H⋯*A*
O2—H2⋯O3^i^	0.82	1.83	2.626 (10)	164

Symmetry code: (i) 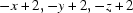.

**Fig. 1.**
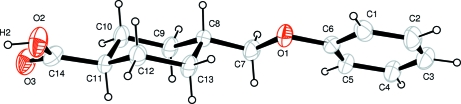
The molecular structure of (I), with displacement ellipsoids drawn at the 30% probability level.

## supplementary crystallographic information

### Comment

To compare the activity of 4-chloromethyl cyclohexane and 4-(tosyloxymethyl)cyclohexane, some cyclohexane derivatives were designed to be linked to substituted phenol. Thus the title compound, a *trans*-4-(phenoxymethyl)cyclohexanecarboxylic acid was synthesized (Sekera & Marvel,1933). We report here the crystal structure of the title compound. The cyclohexane ring of the title compound adopts a chair conformation. The average C—C bond length of the cyclohexane ring is 1.517 (12) Å, is similar to that of *trans*-1,4-cyclohexanedicarboxylic acid (1.523 (3) Å, Luger *et al.*, 1972). The mean endocyclic angle of the cyclohexane is 110.9 (8)°, which is in the range observed for cyclohexane rings (111.4 (4)°, Dunitz & Strickler, 1966).

### Experimental

Methyl *trans*-4-(tosylmethyl)cyclohexanecarboxylate(3.26 g, 10 mmol), phenol(2.82 g, 30 mmol) and potassium phosphate(10.6 g, 50 mmol) were suspended in dry DMF(20 mL) and heated at 368 K for 6 h, then 30 mL water and 30 mL toluene were added to the mixture. The water layer separated was washed twice with toluene and the organic layer combined was washed with water and then dried with sodium sulfate. After filtration and concentration, the crude product was obtained which was further purified by silica gel column chromatography to give pure methyl ester. The ester was hydrolyzed in a mixed solution of 10 mL e thanol and 15 mL 1 N NaOH solution for 5 h at 313 K, after cooling and acidification with hydrochloride the white solid precipitated was collected. Colorless crystals were obtained by slow evaporation in a ethanol-water(4:1) solution at room temperature.

### Refinement

H atoms were positioned geometrically (C—H = 0.93–0.98 Å) and refined using a riding model, with *U*_iso_(H) = 1.2*U*_eq_(C).

### Crystal data

**Table:**

C_14_H_18_O_3_	*F*(000) = 504
*M**_r_* = 234.28	*D*_x_ = 1.205 Mg m^−^^3^
Monoclinic, *P*2_1_/*c*	Mo *K*α radiation, λ = 0.71073 Å
*a* = 6.178 (3) Å	Cell parameters from 30 reflections
*b* = 35.042 (8) Å	θ = 4.5–9.5°
*c* = 6.526 (3) Å	µ = 0.08 mm^−^^1^
β = 113.93 (4)°	*T* = 292 K
*V* = 1291.4 (9) Å^3^	Block, colourless
*Z* = 4	0.45 × 0.25 × 0.24 mm

### Data collection

**Table:**

Enraf–Nonius CAD-4 diffractometer	*R*_int_ = 0.001
Radiation source: fine-focus sealed tube	θ_max_ = 25.5°, θ_min_ = 3.5°
graphite	*h* = −7→6
ω/2–θ scans	*k* = 0→42
2657 measured reflections	*l* = −1→7
2330 independent reflections	3 standard reflections every 250 reflections
1301 reflections with *I* > 2σ(*I*)	intensity decay: 1.8%

### Refinement

**Table:**

Refinement on *F*^2^	Primary atom site location: structure-invariant direct methods
Least-squares matrix: full	Secondary atom site location: difference Fourier map
*R*[*F*^2^ > 2σ(*F*^2^)] = 0.072	Hydrogen site location: inferred from neighbouring sites
*wR*(*F*^2^) = 0.149	H-atom parameters constrained
*S* = 0.97	*w* = 1/[σ^2^(*F*_o_^2^) + (0.0395*P*)^2^] where *P* = (*F*_o_^2^ + 2*F*_c_^2^)/3
2330 reflections	(Δ/σ)_max_ < 0.001
156 parameters	Δρ_max_ = 0.17 e Å^−^^3^
9 restraints	Δρ_min_ = −0.17 e Å^−^^3^

### Special details

**Table:**

Geometry. All e.s.d.'s (except the e.s.d. in the dihedral angle between two l.s. planes) are estimated using the full covariance matrix. The cell e.s.d.'s are taken into account individually in the estimation of e.s.d.'s in distances, angles and torsion angles; correlations between e.s.d.'s in cell parameters are only used when they are defined by crystal symmetry. An approximate (isotropic) treatment of cell e.s.d.'s is used for estimating e.s.d.'s involving l.s. planes.
Refinement. Refinement of *F*^2^ against ALL reflections. The weighted *R*-factor *wR* and goodness of fit *S* are based on *F*^2^, conventional *R*-factors *R* are based on *F*, with *F* set to zero for negative *F*^2^. The threshold expression of *F*^2^ > σ(*F*^2^) is used only for calculating *R*-factors(gt) *etc*. and is not relevant to the choice of reflections for refinement. *R*-factors based on *F*^2^ are statistically about twice as large as those based on *F*, and *R*- factors based on ALL data will be even larger.

### Fractional atomic coordinates and isotropic or equivalent isotropic displacement parameters (Å^2^)

**Table:**

	*x*	*y*	*z*	*U*_iso_*/*U*_eq_	
O1	0.5351 (13)	0.8410 (2)	−0.0641 (11)	0.084 (2)	
O2	1.0653 (15)	0.9709 (3)	0.8119 (13)	0.110 (3)	
H2	1.1166	0.9870	0.9115	0.132*	
O3	0.7287 (14)	0.9889 (2)	0.8198 (11)	0.104 (3)	
C1	0.584 (2)	0.8016 (3)	−0.328 (2)	0.087 (4)	
H1	0.7444	0.8012	−0.2354	0.105*	
C2	0.494 (4)	0.7816 (4)	−0.531 (3)	0.112 (6)	
H2A	0.5971	0.7680	−0.5758	0.134*	
C3	0.260 (4)	0.7818 (4)	−0.662 (3)	0.117 (6)	
H3	0.2030	0.7679	−0.7951	0.141*	
C4	0.103 (3)	0.8026 (4)	−0.6030 (19)	0.102 (5)	
H4	−0.0571	0.8032	−0.6962	0.123*	
C5	0.192 (3)	0.8225 (3)	−0.3991 (19)	0.085 (4)	
H5	0.0893	0.8360	−0.3543	0.102*	
C6	0.425 (3)	0.8221 (3)	−0.268 (2)	0.074 (4)	
C7	0.3845 (19)	0.8640 (3)	0.0058 (16)	0.080 (4)	
H7A	0.2653	0.8483	0.0257	0.096*	
H7B	0.3052	0.8834	−0.1060	0.096*	
C8	0.5426 (19)	0.8826 (3)	0.2262 (15)	0.062 (3)	
H8	0.6310	0.8625	0.3314	0.074*	
C9	0.3865 (17)	0.9033 (3)	0.3227 (15)	0.076 (4)	
H9A	0.2912	0.9223	0.2163	0.092*	
H9B	0.2802	0.8852	0.3460	0.092*	
C10	0.5351 (19)	0.9225 (3)	0.5425 (15)	0.076 (4)	
H10A	0.4323	0.9360	0.5975	0.092*	
H10B	0.6210	0.9033	0.6524	0.092*	
C11	0.7078 (19)	0.9501 (3)	0.5158 (16)	0.066 (3)	
H11	0.6143	0.9689	0.4037	0.079*	
C12	0.8661 (18)	0.9296 (3)	0.4193 (15)	0.075 (3)	
H12A	0.9704	0.9480	0.3942	0.091*	
H12B	0.9635	0.9108	0.5261	0.091*	
C13	0.7164 (19)	0.9100 (3)	0.2005 (16)	0.077 (3)	
H13A	0.6310	0.9292	0.0898	0.092*	
H13B	0.8191	0.8964	0.1460	0.092*	
C14	0.842 (2)	0.9717 (4)	0.7277 (17)	0.073 (4)	

### Atomic displacement parameters (Å^2^)

**Table:**

	*U*^11^	*U*^22^	*U*^33^	*U*^12^	*U*^13^	*U*^23^
O1	0.102 (6)	0.090 (6)	0.065 (5)	−0.001 (5)	0.040 (5)	−0.021 (5)
O2	0.103 (7)	0.143 (9)	0.085 (6)	0.002 (7)	0.040 (6)	−0.046 (5)
O3	0.109 (7)	0.127 (8)	0.083 (6)	0.020 (6)	0.046 (5)	−0.031 (5)
C1	0.120 (12)	0.073 (9)	0.093 (9)	0.000 (8)	0.068 (9)	−0.004 (8)
C2	0.179 (18)	0.097 (12)	0.102 (12)	−0.012 (13)	0.100 (13)	−0.017 (10)
C3	0.20 (2)	0.099 (12)	0.078 (11)	−0.008 (14)	0.078 (13)	−0.007 (9)
C4	0.152 (14)	0.093 (11)	0.070 (9)	−0.011 (10)	0.052 (10)	−0.013 (8)
C5	0.114 (12)	0.088 (10)	0.058 (8)	0.000 (9)	0.038 (8)	−0.009 (8)
C6	0.108 (12)	0.063 (9)	0.063 (8)	0.000 (9)	0.045 (9)	0.002 (7)
C7	0.098 (9)	0.090 (9)	0.066 (7)	−0.006 (8)	0.049 (7)	−0.010 (7)
C8	0.080 (8)	0.056 (8)	0.051 (6)	0.001 (7)	0.029 (6)	−0.004 (6)
C9	0.088 (9)	0.095 (10)	0.061 (7)	−0.013 (7)	0.045 (7)	−0.013 (7)
C10	0.096 (9)	0.090 (9)	0.062 (7)	−0.025 (8)	0.052 (7)	−0.021 (7)
C11	0.084 (9)	0.065 (8)	0.050 (6)	0.005 (7)	0.030 (6)	−0.010 (6)
C12	0.088 (9)	0.079 (9)	0.067 (7)	−0.012 (7)	0.039 (7)	−0.011 (7)
C13	0.089 (9)	0.098 (10)	0.059 (7)	−0.013 (8)	0.044 (7)	−0.017 (7)
C14	0.068 (9)	0.102 (10)	0.055 (7)	0.013 (9)	0.030 (7)	0.004 (7)

### Geometric parameters (Å, °)

**Table:**

O1—C6	1.394 (12)	C7—H7B	0.9700
O1—C7	1.438 (10)	C8—C13	1.501 (12)
O2—C14	1.261 (11)	C8—C9	1.532 (11)
O2—H2	0.8200	C8—H8	0.9800
O3—C14	1.248 (11)	C9—C10	1.512 (12)
C1—C6	1.392 (14)	C9—H9A	0.9700
C1—C2	1.401 (16)	C9—H9B	0.9700
C1—H1	0.9300	C10—C11	1.501 (12)
C2—C3	1.350 (18)	C10—H10A	0.9700
C2—H2A	0.9300	C10—H10B	0.9700
C3—C4	1.386 (17)	C11—C14	1.497 (13)
C3—H3	0.9300	C11—C12	1.540 (12)
C4—C5	1.402 (13)	C11—H11	0.9800
C4—H4	0.9300	C12—C13	1.514 (12)
C5—C6	1.341 (14)	C12—H12A	0.9700
C5—H5	0.9300	C12—H12B	0.9700
C7—C8	1.519 (12)	C13—H13A	0.9700
C7—H7A	0.9700	C13—H13B	0.9700
			
C6—O1—C7	116.2 (9)	C10—C9—H9A	109.4
C14—O2—H2	109.5	C8—C9—H9A	109.4
C6—C1—C2	118.1 (14)	C10—C9—H9B	109.4
C6—C1—H1	120.9	C8—C9—H9B	109.4
C2—C1—H1	120.9	H9A—C9—H9B	108.0
C3—C2—C1	120.5 (16)	C11—C10—C9	111.3 (8)
C3—C2—H2A	119.8	C11—C10—H10A	109.4
C1—C2—H2A	119.8	C9—C10—H10A	109.4
C2—C3—C4	121.1 (16)	C11—C10—H10B	109.4
C2—C3—H3	119.5	C9—C10—H10B	109.4
C4—C3—H3	119.5	H10A—C10—H10B	108.0
C3—C4—C5	118.5 (14)	C14—C11—C10	111.9 (8)
C3—C4—H4	120.7	C14—C11—C12	114.1 (10)
C5—C4—H4	120.7	C10—C11—C12	110.2 (8)
C6—C5—C4	120.3 (12)	C14—C11—H11	106.7
C6—C5—H5	119.8	C10—C11—H11	106.7
C4—C5—H5	119.8	C12—C11—H11	106.7
C5—C6—C1	121.5 (12)	C13—C12—C11	110.5 (9)
C5—C6—O1	125.9 (11)	C13—C12—H12A	109.5
C1—C6—O1	112.7 (13)	C11—C12—H12A	109.5
O1—C7—C8	106.9 (9)	C13—C12—H12B	109.5
O1—C7—H7A	110.3	C11—C12—H12B	109.5
C8—C7—H7A	110.3	H12A—C12—H12B	108.1
O1—C7—H7B	110.3	C8—C13—C12	112.1 (7)
C8—C7—H7B	110.3	C8—C13—H13A	109.2
H7A—C7—H7B	108.6	C12—C13—H13A	109.2
C13—C8—C7	112.5 (8)	C8—C13—H13B	109.2
C13—C8—C9	109.9 (8)	C12—C13—H13B	109.2
C7—C8—C9	108.8 (9)	H13A—C13—H13B	107.9
C13—C8—H8	108.5	O3—C14—O2	122.0 (11)
C7—C8—H8	108.5	O3—C14—C11	118.6 (11)
C9—C8—H8	108.5	O2—C14—C11	119.4 (11)
C10—C9—C8	111.1 (8)		

### Hydrogen-bond geometry (Å, °)

**Table:**

*D*—H···*A*	*D*—H	H···*A*	*D*···*A*	*D*—H···*A*
O2—H2···O3^i^	0.82	1.83	2.626 (10)	164.

Symmetry codes: (i) −*x*+2, −*y*+2, −*z*+2.
